# Evaluation of Antiviral Therapy Performed after Curative Therapy in Patients with HBV-Related Hepatocellular Carcinoma: An Updated Meta-Analysis

**DOI:** 10.1155/2016/5234969

**Published:** 2016-03-10

**Authors:** Peng Yuan, Peng Chen, Yeben Qian

**Affiliations:** Department of Hepatobiliary Surgery, The First Affiliated Hospital of Anhui Medical University, Hefei 230032, China

## Abstract

*Background.* The long-term prognosis after curative therapy for hepatitis B virus- (HBV-) related hepatocellular carcinoma (HCC) remains unsatisfactory due to the high incidence of recurrence. The effect of treatment with nucleotide analogues (NAs) in patients with HBV-related HCC after curative therapy remains unclear.* Objective.* To assess the impact of using NAs after curative therapy.* Method.* A computerized literature search was performed; eligible studies were identified from databases. The pooled risk ratios (RRs) and 95% CIs were calculated using Review Manager 5.3.* Result.* The meta-analysis included a total of 15 studies with 8060 patients. The one-year and three-year recurrence (one-year recurrence: RR 0.41 [95% CI 0.28 to 0.61]; *P* < 0.00001; three-year recurrence: RR 0.63 [95% CI 0.43 to 0.94]; *P* = 0.001) and the one-, three-, and five-year overall survival (OS) and disease-free survival (DFS) were significantly better in the treatment group.* Conclusion.* NAs can reduce the recurrence and improve the prognosis of HBV-related HCC after curative therapy.

## 1. Introduction

Hepatocellular carcinoma (HCC) is the fifth most common cancer in the world and the third leading cause of cancer-related death [1, 2]. It is a common malignancy worldwide, but especially in Asia, due to the endemic status of chronic hepatitis B [3], which is responsible for HBV-related HCC (HBV-HCC). In patients with HBV infection who develop HCC, treatment for one condition often influences the other condition: for example, treatment for HCC may affect viral replication, whereas treatment for chronic HBV infection may influence the clinical outcome of HCC [4]. Curative therapies including liver transplantation, hepatectomy, and local ablative therapy such as radiofrequency ablation are used to treat HCC. With the advances in surgery, the 5-year survival rate after curative therapy has reached 50% [5]. However, this is not satisfactory because the posttreatment recurrence rate is high [6]. Relapse is reported to occur in up to 70% of patients within 5 years of curative treatment [7]. Recent studies have shown that HBV viral replication plays an important role in tumor recurrence and might influence postoperative survival [8–12].

The results of 6 prospective studies, 11 retrospective studies, and 5 meta-analyses have shown that nucleotide analogues (NAs) might improve the overall survival (OS) [4, 8, 10, 13–30]. However, only a few studies have shown that NAs decrease the recurrence rate or improve recurrence-free survival (RFS) [17, 19, 20, 24–26, 29]. Therefore, it is still not clear whether NAs can reduce the recurrence of HCC or improve RFS. In order to [13–25] investigate this, we performed a meta-analysis to sum up the current evidence from the literature, to determine the role that NAs play in the treatment of patients who have undergone curative therapy.

## 2. Method

### 2.1. Study Selection

A computerized search was performed in January 2015 by searching Medline, OVID, Cochrane, Embase, and Chinese Biological Medicine (CBM) from the time of inception to January 2015. The search was performed using the main keywords “HCC”, “liver cancer”, “hepatocellular carcinoma”, “HBV”, “hepatitis B”, “resection”, “hepatectomy”, “curative therapy”, “nucleotide”, “entecavir”, “lamivudine”, “adefovir”, “telbivudine”, “recurrence”, and “prognosis”. Curative therapy was defined as any method that could remove evident tumors completely with no remnant tumor tissue found within 4 weeks after the treatment.

We included randomized controlled trials (RCTs), prospective cohort trials, and retrospective cohort trails that (1) included patients with confirmed chronic HBV infection; (2) included patients with confirmed HCC as determined by histopathological examination or radiological examination in addition to alpha-fetoprotein; (3) used postoperative antiviral treatment; (4) did not administer any treatment for control patients and those with HCC recurrence and/or mortality that developed during the follow-up period before the patients were excluded from the trials; and (5) included the OS, disease-free survival (DFS), or recurrence rate (one of the three results) in the follow-up data (studies that included RFS were also accepted as RFS is considered equivalent to DFS).

We excluded (1) studies on patients who received antiviral therapy before HCC diagnosis; (2) studies in which noncurative therapies including image-guided tumor ablation using chemical or thermal ablative techniques for unresectable tumors and transarterial chemoembolization (TACE) were performed; (3) studies that included patients coinfected with HCV, HDV, or HIV; (4) studies on patients with daily alcohol consumption; and (5) studies on patients with drug abuse.

### 2.2. Data Extraction and Validity Assessment

The methodological qualities and relevance of the studies were assessed by two reviewers (Peng Yuan and Peng Chen) independently. The following data were extracted by the two reviewers: type of study, year of study, sample size, characteristics of the patients, follow-up duration, tumor stage, HBV DNA level, Child-Pugh grade, ALT level, cirrhosis rate, tumor characteristics, types of curative treatment, tumor recurrence rate, DFS (or RFS), and OS. The quality of studies was assessed for RCT studies and cohort studies using the JADAD scale [31] and Newcastle-Ottawa scale [32], respectively (Table 1). If any discrepancies were noticed between the two reviewers in the process of data extraction and quality assessment, a third reviewer (Yeben Qian) made the final decision.

### 2.3. Statistical Analysis

RevMan version 5.3 was used to perform the statistical analyses. Relative risk (RR) was determined for each study. A random-effect model was used in this meta-analysis to calculate the overall effects estimates. Heterogeneity was assessed based on the *I* ^2^ value, which indicated the percentage of total variation across studies and *P* < 0.1 was considered to indicate statistical significance. The funnel plot was used to evaluate publication biases.

## 3. Results

### 3.1. Study Selection and Characteristics of the Included Studies

**Figure 1:**
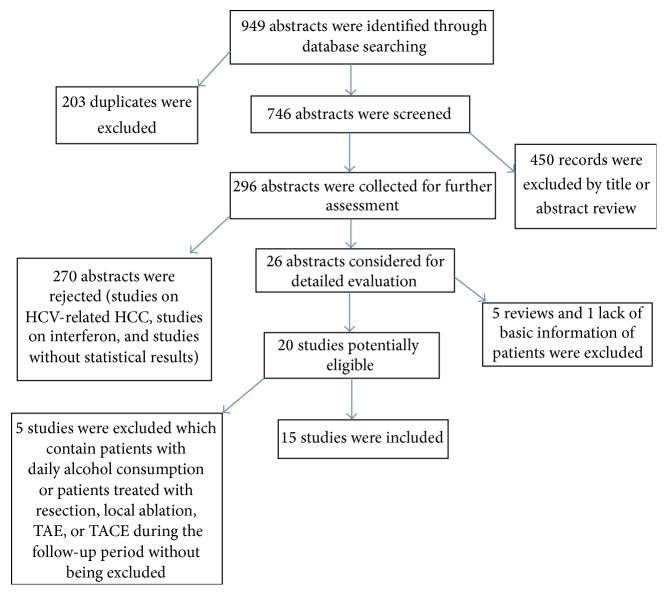
Flow chart depicting the study selection process: 15 studies were included in this meta-analysis.

The literature search yielded 949 abstracts. As showed in Figure 1, by excluding 203 duplicates and 450 irrelevant abstracts, 296 were collected for further evaluation, of which 26 abstracts were considered for detailed evaluation and the remaining 270 were rejected because they were on HCV-related HCC or interferon therapy, they did not provide the statistical results required, or they were on patients who did not receive curative therapy before the use of NAs. Among the 26 publications, five meta-analyses and one study that lacked basic information on patients were excluded, and five other studies were finally excluded because one of them included patients with daily alcohol consumption and the other four did not exclude patients who had undergone resection, local ablation, transarterial embolization (TAE), or TACE during the follow-up period. Finally, 15 studies were included in the meta-analysis, including two RCTs and 13 cohort studies (three prospective studies, nine retrospective studies, and one that had both prospective and retrospective samples). In the study by Yin et al. [20], a randomized controlled trial and a nonrandomized trial were performed, so we considered this as two individual studies in this meta-analysis.

This analysis included a total of 8060 patients, 2498 of whom were in the treatment group and 5562 of whom were in the control group (without NA treatment). The curative therapies performed included liver resection; local ablation; and all treatments which guaranteed that all macroscopically evident tumors were removed completely, tumor cells were not present along the parenchymal transection line (which was confirmed histologically), and computed tomography performed at least four weeks after surgery did not show any remnant tumor tissue. Baseline comparison was performed in all the 15 studies, and no significant difference was found between both groups in terms of age, gender ratio, Child-Pugh grade, percentage of HBeAg-positive patients, HBV DNA level, presence of cirrhosis, tumor stage, AFP level, and HCC treatment. Among the 15 studies included, nine studies used curative hepatectomy as the initial treatment for HCC while the other five studies included patients treated with radiofrequency ablation and other curative therapies. Four studies used single-nucleotide analogues as antiviral therapy (two used adefovir and two used lamivudine), and the other 11 studies initiated the treatment with lamivudine or entecavir and then switched to adefovir, telbivudine, or tenofovir disoproxil fumarate when drug resistance occurred or TMDD mutants were detected.

### 3.2. Antiviral Treatment and Recurrence

**Figure 2:**
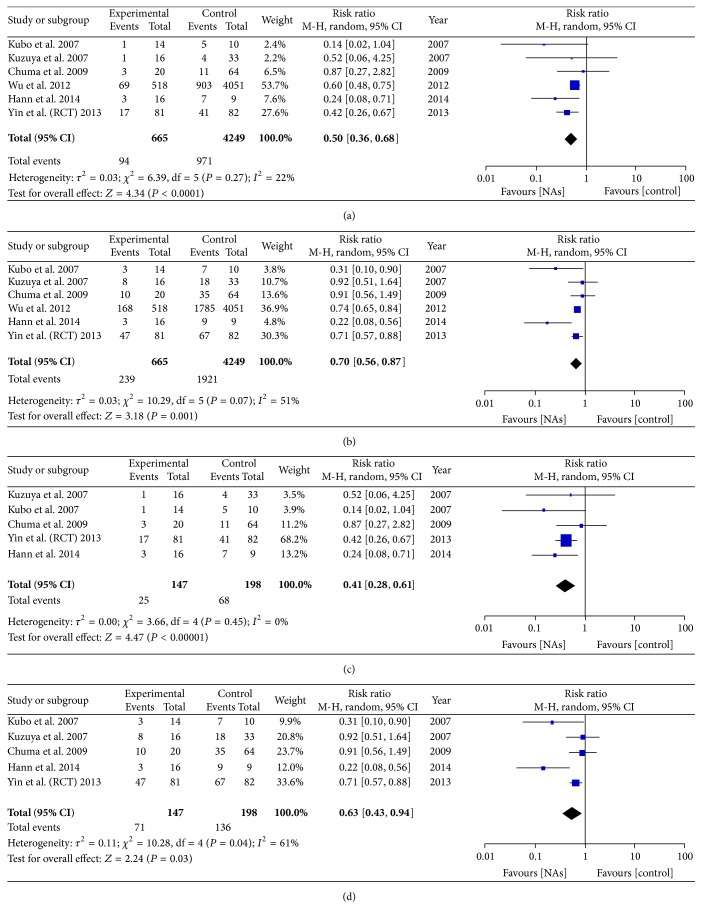
(a) Forest plot showing the impact of NAs on the 1-year recurrence rate without Wu et al.'s study. (b) Forest plot showing the impact of NAs on the 3-year recurrence rate without Wu et al.'s study. (c) Forest plot showing the impact of NAs on the 1-year recurrence rate with Wu et al.'s study. (d) Forest plot showing the impact of NAs on the 3-year recurrence rate with Wu et al.'s study.

We pooled the data from 6 of 15 studies which reported the recurrence rate or cause of death of patients, so that we could calculate the number of patients with HCC recurrence. However, in Wu et al.'s [25] study, a large number of patients who used drugs, including NSAIDs and statins, and patients with diabetes were not excluded from the retrospective cohorts. As the number of samples in which recurrence was analyzed was not sufficient, Wu et al.'s study was included and analyzed. We found that the one-year recurrence rate was significantly lower in the antiviral treatment group (94 of 665) than in the untreated group (971 of 4249), with the risk of recurrence reduced by 50% in the treatment group (RR = 0.50, 95% CI = 0.36–0.68, *P* < 0.0001, Figure 2(a)). The three-year recurrence rate was also lower in the antiviral treatment group (239 of 665) than in the untreated group (1921 of 4249). The risk of recurrence was found to be reduced by 30% (RR = 0.70, 95% CI = 0.56–0.87, *P* = 0.001, Figure 2(b)) on pooling the data from all the six studies. There was no significant heterogeneity in the 1-year while there was heterogeneity in 3-year recurrence rate (1-year recurrence: *I* ^2^ = 22% and *P* = 0.27; 3-year recurrence: *I* ^2^ = 51% and *P* = 0.07). The funnel plot for evaluation of publication bias is shown in Figure 5.

When Wu et al.'s study was not included, the 1-year recurrence rate was significantly lower in the antiviral treatment group (25 of 147) than in the untreated group (68 of 198), with the risk of recurrence reduced by 59% in the treatment group (RR = 0.41, 95% CI = 0.28–0.61, *P* < 0.00001, Figure 2(c)). Further, the 3-year recurrence rate was also lower in the antiviral treatment group (71 of 147) than in the untreated group (136 of 198). The risk of recurrence was found to be reduced by 37% (RR = 0.63, 95% CI = 0.43–0.94, *P* = 0.03, Figure 2(d)) on pooling the data from the five studies (after excluding Wu et al.'s study). There was no significant heterogeneity in the 1-year while there was heterogeneity in 3-year recurrence rate (1-year recurrence: *I* ^2^ = 0% and *P* = 0.45; 3-year recurrence: *I* ^2^ = 61% and *P* = 0.04).

### 3.3. Antiviral Treatment and OS

**Figure 3:**
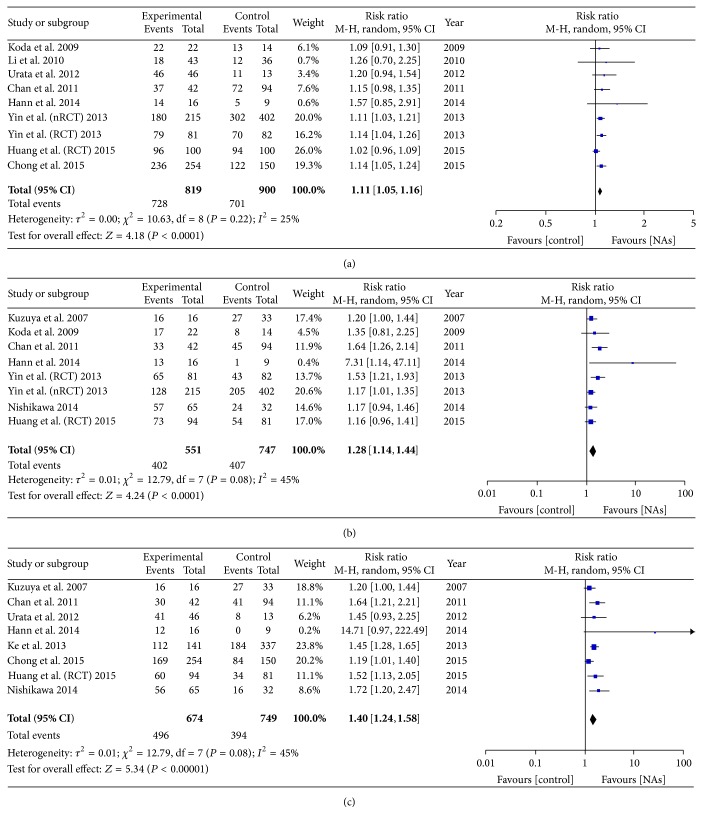
(a) Forest plot showing the impact of NAs on the 1-year OS. (b) Forest plot showing the impact of NAs on the 3-year OS. (c) Forest plot showing the impact of NAs on the 5-year OS.

On pooling the data from 10 of 15 studies, the 1-year OS was found to be higher in the antiviral treatment group (728 of 819) than in the untreated group (701 of 900), with an improvement of 11% in the treatment group (RR = 1.11, 95% CI = 1.05–1.16, *P* < 0.0001, Figure 3(a)). Further, the 3-year and 5-year OS values were significantly higher in the antiviral treatment group (402/551 and 496/674, resp.) than in the untreated group (407/747 and 394/749, resp.), which corresponded to an improvement of 28% (RR = 1.28; 95% CI = 1.14–1.44, *P* < 0.0001, Figure 3(b)) and 40% (RR = 1.40, 95% CI = 1.24–1.58, *P* < 0.00001, Figure 3(c)), respectively. Heterogeneity was observed in the 3-year and 5-year OS, but no significant heterogeneity observed in 1-year OS (1-year OS: *I* ^2^ = 25% and *P* = 0.22; 3-year OS: *I* ^2^ = 45% and *P* = 0.08; 5-year OS: *I* ^2^ = 45% and *P* = 0.08).

### 3.4. Antiviral Treatment and DFS/RFS

**Figure 4:**
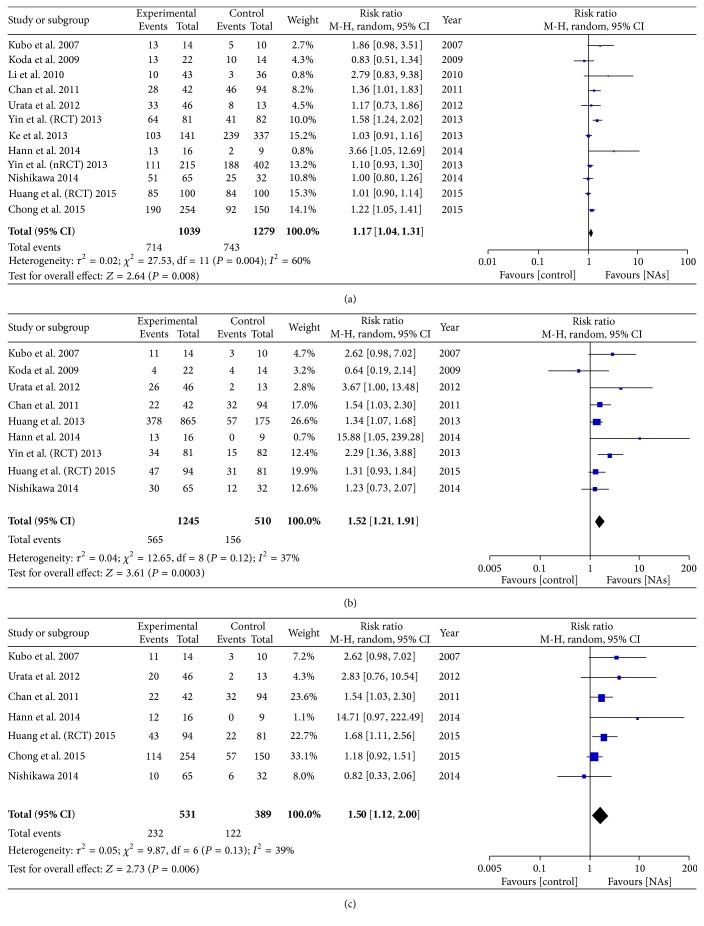
(a) Forest plot showing the impact of NAs on the 1-year DFS. (b) Forest plot showing the impact of NAs on the 3-year DFS. (c) Forest plot showing the impact of NAs on the 5-year DFS.

**Figure 5:**
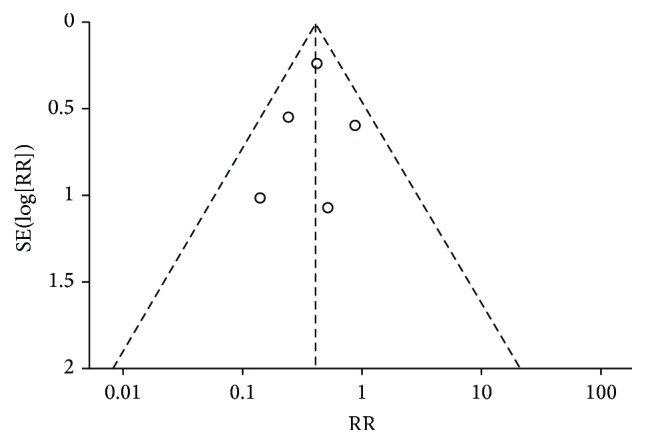
Funnel plot analysis of publication bias. The outcome impact of NAs on the 1-year HCC recurrence rate.

On pooling the data from 12 of 15 studies, we found that the 1-year DFS was higher in the antiviral treatment group (714 of 1093) than in the untreated group (743 of 1279), and DFS improved by 17% with antiviral treatment (RR = 1.17, 95% CI = 1.04–1.31, *P* < 0.008, Figure 4(a)). Further, the 3-year and 5-year DFS values were significantly higher in the antiviral treatment group (555/1245 and 232/531, resp.) than in the untreated group (156/510 and 122/389, resp.), which means that DFS improved by 52% (RR = 1.52, 95% CI = 1.21–1.91, *P* = 0.0003, Figure 4(b)) and 50% (RR = 1.50, 95% CI = 1.12–2.00, *P* = 0.006, Figure 4(c)), respectively, with antiviral treatment. Low heterogeneity was observed in the 3-year and 5-year DFS (3-year DFS: *I* ^2^ = 37% and *P* = 0.12; 5-year DFS: *I* ^2^ = 39% and *P* = 0.13), but median heterogeneity was observed in the 1-year DFS (*I* ^2^ = 60%, *P* = 0.004).

### 3.5. Subgroup Analysis with RCTs Excluded and High Score Studies

In subgroups, we pooled data from all observational studies and studies with Newcastle-Ottawa no less than 8 stars, respectively. The results still showed significant difference between NAs group and untreated group. The result was shown in Table 2.

## 4. Discussion

Analysis of the data from the 15 studies examined provided significant evidence of the benefits of using NAs, as the use of NAs was associated with a decreased HCC recurrence rate after curative therapy and improved RFS, especially long-term survival (more than one year). HBV-related HCC accounts for the majority of HCC cases in the Asia-Pacific region: 80–90% of patients with HCC have chronic HBV infection [26]. With the advances in surgery, the survival rate of HBV-related HCC after curative therapy has improved, but the recurrence rate is still high and severely affects patient survival. The mechanism of HCC recurrence is still unclear; however, the results of different types of studies (RCTs, cohort studies, and meta-analyses) have indicated that a high viral load of HBV before or after curative treatment may increase the recurrence rate of patients after curative surgery for HCC and reduce OS and DFS [13, 20, 27, 28].

The use of NAs, including lamivudine, adefovir dipivoxil, and entecavir, has been proved to be beneficial in preventing progression to cirrhosis and delaying the development of HCC in patients with chronic HBV infection [33, 35–39]. Compared with other adjuvant therapies, especially interferon therapy, NA therapy has been shown to be safer and better tolerated; it has been shown that NA therapy can reduce the risk of hepatic decompensation with life-threatening complications, including hepatic encephalopathy, icterus, ascites, and variceal bleeding, in patients with advanced liver disease [40]. However, the effects of NAs in patients who have undergone initial curative therapy are unclear for many reasons. (1) One of the reasons is the lack of enough samples, as it is difficult to perform RCTs for ethical reasons. (2) Another reason is the emergence of drug resistance, as evidenced by the rate of lamivudine resistance, which has been reported to be 14–39% [16, 17, 21, 22]. Although newer NAs that are currently recommended by international guidelines may provide better viral suppression and potentially even better long-term outcomes [15, 41–43], in rural areas and underdeveloped regions, lamivudine is the only drug that patients wish to take for economic reasons. (3) Yet another reason is that it is difficult to eliminate differences between surgeons and hospitals with regard to the treatment strategies. (4) Finally, as patients with HBV-related HCC often have other serious diseases such as peptic ulcer, hypertension, and renal dysfunctions, which would have an influence on the effect of NAs, it would be difficult to understand the true effect of NAs in these patients.

Our meta-analysis tried to eliminate the abovementioned limitations as much as possible and come to a relatively objective conclusion. The results showed that the use of NAs to treat patients after curative therapy for HBV-related HCC might reduce recurrence and improve the OS and DFS or RFS.

The short-term recurrence rate (1-year and 3-year) was obviously higher in the untreated groups, but we were unable to determine the recurrence rate over a longer period because of the unavailability of raw data and detailed information on the causes of death. For example, in cases where death was caused by liver failure, it would be very difficult to separate the role of the tumor from the role of liver failure and therefore determine the exact recurrence rate. Therefore, we only included studies in which the recurrence rates were already calculated. OS was higher in the treatment groups at 1, 3, and 5 years, but the 3- and 5-year OS values were more obviously higher in the treatment group. This finding could be attributable to the advances in surgical treatment for HBV-related HCC and overall improvement in short-term prognosis. Analysis of DFS revealed similar results.

As our meta-analysis revealed, NAs can reduce the recurrence of HCC and improve DFS after curative therapy, which is in contrast to the conclusion of some studies that NAs do not reduce the recurrence rate [4, 16, 18, 22, 23, 27]. Furthermore, we tried to eliminate the effects of NSAIDs by excluding studies in which HCC patients were given these drugs, because latest research has revealed that NSAIDs might also reduce the recurrence of HCC after curative therapy [44]. Further, the 1-year and 3-year recurrence rates considerably improved with or without the inclusion of Wu et al.'s study.

There are several limitations in our meta-analysis: (1) In the 11 cohort studies included, the treatment and no-treatment groups were not matched. (2) The cut-off value of serum HBV DNA in the trials differs: although the majority of the references set the cut-off value as 10^4^ copies/mL, the cut-off value was 500 copies/mL in Yin et al.'s study [20], and four other studies [4, 8, 18, 26] included samples in which the serum HBV level was lower than 10^4^ copies/mL. (3) The heterogeneity of the baseline characteristics, such as tumor size, number of tumors, and cirrhosis rate before initial HCC treatment, may cause a potential bias. (4) In prospective studies, it was difficult to exclude samples in which the patients were not followed up, which may have caused a potential bias. (5) Some of the studies did not exclude patients who used NSAIDs during the follow-up period, so we need to further investigate this by eliminating the effect of NSAIDs. (6) Although liver transplantation is one of most important curative therapies on HCC, unfortunately no applicable study on using NAs after liver transplantation was included in our meta-analysis.

In conclusion, antiviral therapy with NAs has potential benefits with regard to reducing the recurrence rate and improving the OS and DFS of patients with HBV-related HCC after curative therapy. Therefore, NA treatment should be recommended for individuals with HBV-related HCC, provided their serum HBV level and general health status are suitable for NA use.

## Figures and Tables

**Table 1:** Characteristics and quality of studies comparing treatment against no treatment in HCC recurrence.

Study	Number of Patients^a^	Sex (M/F)^c^	Age^c^	Tumor stage^b^	Multiple tumors (*n*)^c^	Presence of portal vein tumor thrombus (*n*)^c^	Tumor capsule completed/uncompleted (*n*)^c^	Tumor size (cm)^c^	HBV DNA level (log copies/mL)^c^	ALT (IU/L)^c^	Cirrhosis (*n*)^c^	Type of treatment	Follow-up duration (months)^c^	Quality score
Li et al. 2010 [16]	79 (43 versus 36)	34/9 versus 30/6	46 versus 45	TNM: 9/17/17 versus 4/10/12	N/A	10 versus 13	N/A	7.1 versus 8.5	N/A	N/A	24 versus 25	Resection	12 versus 12	6^e^
Kubo et al. 2007 [17]	24 (14 versus 10)	10/4 versus 7/3	55 versus 55	TNM: 2/7/5 versus 3/2/5	5 versus 5	4 versus 4	N/A	2.4 versus 2.8	N/A	53 versus 56	N/A	Resection	37 versus 7	7^e^
Chuma et al. 2009 [24]	84 (20 versus 64)	14/6 versus 50/14	56 versus 56	TNM: 9/10/1 versus 22/34/8	5 versus 16	N/A	N/A	1.7 versus 2.0	N/A	43 versus 30	11 versus 31	Resection and RFA	34 versus 53	8^e^
Kuzuya et al. 2007 [22]	49 (16 versus 33)	14/2 versus 27/6	60 versus 61	TNM: 12/3/1 versus 13/16/4	N/A	N/A	N/A	N/A	6.2 versus 4.1	57 versus 54	N/A	Resection and RFA	38 versus 33	7^e^
Koda et al. 2009 [21]	36 (22 versus 14)	24/6 versus 15/5	59 versus 60	TNM: 19/20/11/0	N/A	N/A	N/A	N/A	N/A	78 versus 54	N/A	RFA, PEI, and TAE	59	7^e^
Hann et al. 2014 [26]	25 (16 versus 9)	14/2 versus 16/0	57 (20–73) versus 53 (46–60)	N/A	0/0	N/A	N/A	2.65 (1–7) versus 3 (1–6.9)	5.4 (2.7–8.4) versus 6.9 (2.9–7.2)	N/A	N/A	Local ablation	53 (48–66) versus 57 (20–73)	9^e^
Ke et al. 2013 [27]	478 (141 versus 337)	129/12 versus 284/53	49 versus 50	BCLC: 107/23/11 versus 256/60/21	39 versus 97	N/A	84/57 versus 163/174	N/A	N/A	39 (2–504) versus 35 (2–341)	82 versus 79	Resection	24 (2–65) versus 24 (0–73)	6^e^
Huang et al. 2013 [8]	1040 (86 versus 175)	758/107 versus 156/19	51 versus 52	N/A	N/A	N/A	N/A	5.2 versus 5.3	N/A	58 versus 48	N/A	Resection	42 (4–72)	7^e^
Yin et al. 2013 [20]	617 (215 versus 402)	194/21 versus 336/66	50 versus 50	BCLC: 6/159/50 versus 6/296/98	31 versus 51	30 versus 62	87/128 versus 158/244	N/A	4.51 versus 3.82	N/A	101 versus 144	Resection	24 (median)	8^e^
Yin et al. 2013 [20]	163 (81 versus 82)	74/7 versus 70/12	48 versus 49	BCLC: 4/67/10 versus 2/58/22	10 versus 18	3 versus 6	38/43 versus 22/50	N/A	4.88 versus 4.57	N/A	20 versus 23	Resection	40 (median)	3^d^
Urata et al. 2012 [28]	59 (46 versus 13)	34/12 versus 8/5	57 versus 58	N/A	8 versus 13	N/A	N/A	2.8 versus 3.4	4.7 versus 6.1	47 versus 58	21 versus 4	Resection	37 (2–132)	7^e^
Chan et al. 2011 [33]	136 (42 versus 94)	31/11 versus 74/20	57 (36–83) versus 55 (22–81)	AJCC: 11/14/16 versus 28/18/48	N/A	N/A	N/A	9.3 (1–28) versus 9.0 (2–21)	N/A	58 (12–182) versus 43 (13–393)	31 versus 53	Resection	N/A	7^e^
Chong et al. 2015 [4]	404 (254 versus 150)	222/32 versus 125/25	56 (32–80) versus 55 (27–81)	AJCC: 16/50/34/2 versus 97/27/26/0	56 versus 32	N/A	N/A	N/A	N/A	43 (10–2452) versus 44 (14–262)	168 versus 79	Resection	39 (0–164) versus 43 (0–151)	8^e^
Huang et al. 2015 [19]	200 (100 versus 100)	90/10 versus 89/11	50 versus 51	BLCL (0/A): 8/60 versus 8/59	N/A	N/A	70/30 versus 69/31	4.9 vs/5.1	N/A	53 versus 51	N/A	Resection	60 (4–70)	4^d^
Wu et al. 2012 [25]	4569 (518 versus 4051)	435/83 versus 3335/716	54 versus 55	N/A	N/A	N/A	N/A	N/A	N/A	N/A	252 versus 1568	Resection	32 versus 26	8^e^
Nishikawa et al. 2014 [34]	97 (65 versus 32)	47/18 versus 20/12	56 versus 61	TNM: 16/33/16 versus 7/16/9	25 versus 9	N/A	N/A	2.8 versus 3.2	N/A	53 versus 40	38 versus 15	RFA and PEI	59 versus 48	7^e^

M: male; F: female; ALT: alanine aminotransferase; N/A: not available; randomized controlled trials were overstriking; RFA: radiofrequency ablation; PEI: percutaneous ethanol injection; TAE: transcatheter arterial embolization.

^a^Patient with treatment versus no treatment.

^b^Tumor stage containing TNM, AJCC with form (I/II/III or I/II/III/IV), and BCLC with form (0/A/B).

^c^Average or median (range).

^d^JADAD scale (0–5).

^e^Newcastle-Ottawa scale (stars).

**Table 2:** Summary of the results of subgroup analysis between the two groups according to NOS scale and type of study.

Variables	Num.	NA group, events/total	Control group, events/total	RR (95% CI)	*P* value	Cross-study heterogeneity			
						*χ* ^2^	df	*I* ^2^ (%)	*P* value
Studies with no less than 8 stars according to Newcastle-Ottawa scale									
1-year recurrence	3	75/554	921/4124	0.54 [0.32, 0.91]	0.02	3.09	2	35	0.21
3-year recurrence	3	181/554	1829/4124	0.63 [0.38, 1.05]	0.08	7.18	2	72	0.03
1-year overall survival	3	430/485	429/561	1.13 [1.07, 1.20]	<0.0001	1.31	2	0	0.52
3-year overall survival	2	141/231	206/411	2.32 [0.39, 13.89]	0.36	3.92	1	74	0.052
5-year overall survival	3	181/270	84/159	2.99 [0.24, 34.42]	0.40	3.72	1	73	0.05
1-year disease-free survival	3	314/485	282/561	1.19 [1.09, 1.44]	0.07	3.99	2	50	0.14
5-year disease-free survival	2	126/270	57/159	2.96 [0.24, 36.31]	0.40	3.64	1	73	0.06
Studies with RCTs excluded									
1-year recurrence	5	77/584	930/4167	0.51 [0.33, 0.80]	0.003	4.99	4	20	0.29
3-year recurrence	5	192/584	1854/4167	0.65 [0.44, 0.95]	0.03	10.25	4	61	0.04
1-year overall survival	7	553/638	537/718	1.13 [1.08, 1.19]	<0.00001	1.95	6	0	0.92
3-year overall survival	6	264/376	310/584	1.27 [1.10, 1.47]	0.001	9.25	5	46	0.10
5-year overall survival	7	436/580	360/668	1.39 [1.21, 1.59]	<0.00001	12.19	6	51	0.06
1-year disease-free survival	10	565/585	618/1097	1.14 [1.02, 1.28]	0.02	15.92	9	43	0.07
3-year disease-free survival	7	484/1070	110/347	1.49 [1.11, 1.99]	0.008	9.20	6	35	0.16
5-year disease-free survival	6	189/437	100/308	1.45 [1.02, 2.13]	0.04	8.83	5	43	0.12

Num.: number; RR: risk ratio; CI: confidence interval; df: degrees of freedom.
